# Effectiveness, acceptability, and feasibility of naloxone in carceral settings: a scoping review

**DOI:** 10.1186/s12954-025-01358-x

**Published:** 2025-11-29

**Authors:** Nandakumar Ravichandran, Walter Cullen, Marie Claire Van Hout, Jennifer Smyth, Des Crowley

**Affiliations:** 1 School of Medicine, University College Dublin, Dublin, Ireland; 2 South East Technological University, Waterford, Ireland; 3 HSE Addiction Services, National Social Inclusion Office, Dublin, Ireland; 4 Irish College of General Practitioners, Dublin, Ireland

**Keywords:** Opioid users, Naloxone, Carceral, Incarcerated, Prison

## Abstract

**Background:**

Opioid dependence is highly prevalent within incarcerated populations, with one-fifth of all drug overdose deaths occurring among formerly incarcerated individuals. Opioid antagonists are promising in reversing overdoses, with naloxone widely recognised as a rapid and safe treatment for opioid toxicity. While community-based evidence demonstrates the efficacy of naloxone in reducing overdose deaths, its implementation in carceral settings is neither standardised nor embedded.

**Objective:**

This scoping review aims to systematically assess the acceptability, feasibility, and effectiveness of naloxone interventions in prisons and other detention settings globally, with the explicit goal of identifying research gaps and generating evidence to inform global prison policy, practice and future implementation research.

**Methods:**

A scoping review was conducted following Arksey and O’Malley’s framework. Literature searches were performed in PubMed, Scopus, and Embase for English-language records published between 2000 and 2025, in line with the PRISMA Extension for Scoping Reviews guidelines. Data were analysed using narrative synthesis as informed by Popay et al.

**Results:**

Of the 1764 records initially identified, 24 records met the inclusion criteria. Three themes and associated sub-themes were identified. They were (a) current provision (naloxone distribution and benefits; Take-Home Naloxone programmes on community release; naloxone (intranasal) within carceral settings; rollout of naloxone vending machines), (b) population satisfaction (acceptability and feasibility; implementation readiness) and (c) organisational factors (barriers and challenges; facilitators and suggestions).

**Conclusion:**

Naloxone provision in carceral settings is acceptable, feasible, and effective in preventing opioid overdoses during incarceration and after release. Strong support exists among prisoners and staff, yet significant gaps remain, particularly regarding use in vulnerable populations such as women, younger individuals, and immigration detainees. Integrating naloxone into prison health systems, alongside staff training and peer engagement, is a critical step in reducing preventable overdose deaths and enhancing continuity of care post-release.

## Introduction

Opioid use is a significant public health issue within incarcerated populations, with individuals in prison exhibiting disproportionately high rates of opioid use disorders (OUD) compared to the general population [1]. One of the most alarming consequences of this disparity is the increased risk of fatal opioid-related overdose following release from prison, among people with reduced opioid tolerance due to a period of abstinence during incarceration [2]. This risk is especially high among people who inject drugs (PWIDs), individuals engaged in polydrug use, and those undiagnosed or untreated for drug and alcohol dependence [2–6]. Notably, nearly one-fifth of all drug overdose deaths occur among formerly incarcerated individuals, highlighting both a critical window of vulnerability in the immediate post-release period and an opportunity for public health interventions [1, 5, 7].

The opioid crisis continues to escalate globally, with rising mortality rates driven largely by the increased availability and potency of opioids, including synthetic opioids such as fentanyl, rather than heroin [8, 9]. In the United States, while heroin‑involved overdose deaths declined by 33% between 2022 and 2023, the broader opioid‑involved mortality rate remains high and is increasingly driven by synthetic opioids [10]. In this context, opioid antagonists have emerged as vital tools in the emergency management of opioid overdoses [11, 12]. These agents function by binding to opioid receptors in the central and peripheral nervous systems, thereby reversing the effects of opioids [12]. Two commonly used opioid antagonists are naloxone and naltrexone [13]. While naltrexone is primarily indicated for the treatment of alcohol and opioid dependence in maintenance therapy, naloxone is widely recognised for its rapid action in temporarily reversing opioid toxicity. With a half-life of 30 to 80 min, naloxone may require repeated administration if long-acting opioids (e.g., methadone, fentanyl, buprenorphine) are involved. Individuals should be observed until the risk of recurrent respiratory depression has passed, with readiness to provide additional doses if needed, consistent with standard overdose-response training in community and custodial settings [12, 14].

Marketed under the brand names ‘Narcan’ and ‘RiVive’ in the United States, naloxone is approved by the Food and Drug Administration (FDA) and is available in various formulations, including intravenous, intramuscular, and intranasal routes [13]. Other brand names include ‘Nyxoid’ and the ‘Pebble’ (intranasal) and ‘Prenoxad’ (injectable) in the United Kingdom, ‘Ventizolve’ (intranasal) in Norway. In Ireland, naloxone is available under the names ‘Nyxoid’ and ‘Ventizolve’ (intranasal) and ‘Prenoxad’ (injectable). Studies have reported high naloxone acceptability (97.8%) and carriage rates (61.1%), among PWIDs and considerable over-the-counter carriage rates in pharmacies (38.3%) [15, 16]. Zang et al. highlighted that a supply-based approach to naloxone could reduce overdose deaths by about 6.3%, a demand-based approach could achieve an 8.8% reduction, and combining naloxone distribution with interventions addressing solitary drug use could lead to a reduction in opioid overdose deaths by up to 37.4% [17].

The Penal Reform International’s (PRI) Global Prison Trends Report published in 2025 documented a 27% increase in the global prison population over the past 25 years, slightly below the world population growth of 31%, estimating that 11.5 million people are held in prison worldwide on any given day [18]. Europe experienced a 12% increase over the same period, with notable regional differences in prosecution and conviction rates [18]. It also has the highest incarceration rates for both drug trafficking and drug use/possession [18]. These figures highlight the scale and complexity of carceral settings globally and underscore the importance of understanding harm reduction interventions in this context [18].

Globally many individuals lack access to opioid substitution treatment (OST) while incarcerated and on release leading to increase overdose risk due to lowered opioid tolerance and lack of treatment support [5, 19]. Similarly, other European studies have reported elevated opioid overdose risk in incarcerated men in their late 30 s, often involving polydrug use and absence from substitution treatments [20–22].

During the 67th session of the United Nations Commission on Narcotic Drugs (CND), discussions on ‘Drug use, harm reduction and the right to health’ underscored the necessity of harm reduction strategies, including in carceral settings [23]. The CND also adopted a resolution recognising harm reduction as an essential component of an effective public health response to the non-medical use of illicit drugs [24]. Despite these developments, harm reduction measures in prisons remain limited, emphasizing the need for systematic investigation of interventions like naloxone distribution in correctional facilities worldwide [23]. Given the growing global prison population and the international emphasis on harm reduction including CND commitments and PRI recommendations, there is a clear need to systematically examine naloxone interventions in carceral settings worldwide including access to naloxone for prison staff, prisoners and provision of naloxone on release.

In Ireland, approximately 70% of individuals entering the prison have a history of drug use [25]. In 2021, there were 354 drug-related overdose deaths, 64% male and 36% female, with median ages of 41 years and 47 years respectively [26]. Opioids were the most commonly implicated drug group (69%), with poly-drug use reported in 80% of cases; among those who died alone, opioids were implicated in 70% of deaths [26].

Despite the availability of prison-based health programmes internationally and the well-established effectiveness of naloxone in emergency and community settings, its implementation within carceral settings in Ireland and globally remains neither standardised nor fully embedded [27–30]. By examining global evidence, this review seeks to address these gaps and provide insights that are internationally relevant.

This scoping review is the first to assess the acceptability, feasibility, and effectiveness of naloxone within carceral settings globally, with the explicit aim of identifying research gaps and generating evidence to inform prison policy, practice, and future implementation research.

## Methods

This scoping review was conducted in July 2025. The review framework followed Arksey and O’Malley’s six-step methodological framework with additional recommendations provided by Levac et al. [31, 32]. This framework involves identifying the research question, conducting comprehensive search, selecting relevant records, charting the data, and summarising the findings.

### Stage 1: identifying the research question

This scoping review aimed to explore the acceptability, feasibility and effectiveness of naloxone in carceral settings. The term ‘acceptability’ here refers to how well naloxone programmes are perceived as appropriate or satisfactory by staff or incarcerated individuals, including attitudes, willingness to participate, and perceived value, ‘feasibility’ refers to whether naloxone programmes can be implemented successfully within prisons, considering resources, staff capacity, logistics, and institutional support and ‘effectiveness’ relates to outcomes such as overdose prevention, reversal, or reductions in overdose deaths.

The following research question was formulated:

“What is the acceptability, feasibility and effectiveness of naloxone in carceral settings?”

### Stage 2: identifying relevant records

A literature search was carried out on PubMed, Embase, and Scopus databases. Several sources that met the predefined inclusion criteria were incorporated from other sources to ensure a comprehensive review. Searches covered publications from 2000 to 2025 and were limited to English. The search syntax is detailed below.

**Table:** 

(("Naloxone"[Mesh] OR naloxone[Tiab] Nyxoid[Tiab] OR Prenoxad[Tiab] OR Narcan[Tiab] OR Evzio[Tiab] OR Kloxxado[Tiab] OR Zimhi[Tiab]) AND (prison*[Tiab] OR jail*[Tiab] OR incarcerat*[Tiab] OR correctional[Tiab] OR detainee*[Tiab] OR detention[Tiab] OR carceral[Tiab] OR "post-release"[Tiab] OR "take-home"[Tiab]) AND (treat*[Tiab] OR therap*[Tiab] OR outcome*[Tiab] OR access*[Tiab] OR implement*[Tiab] OR interven*[Tiab] OR "overdose prevention"[Tiab] OR acceptab*[Tiab] OR feasib*[Tiab] OR effic*[Tiab] OR impact[Tiab]))	

### Stage 3: study selection

**Fig. 1:**
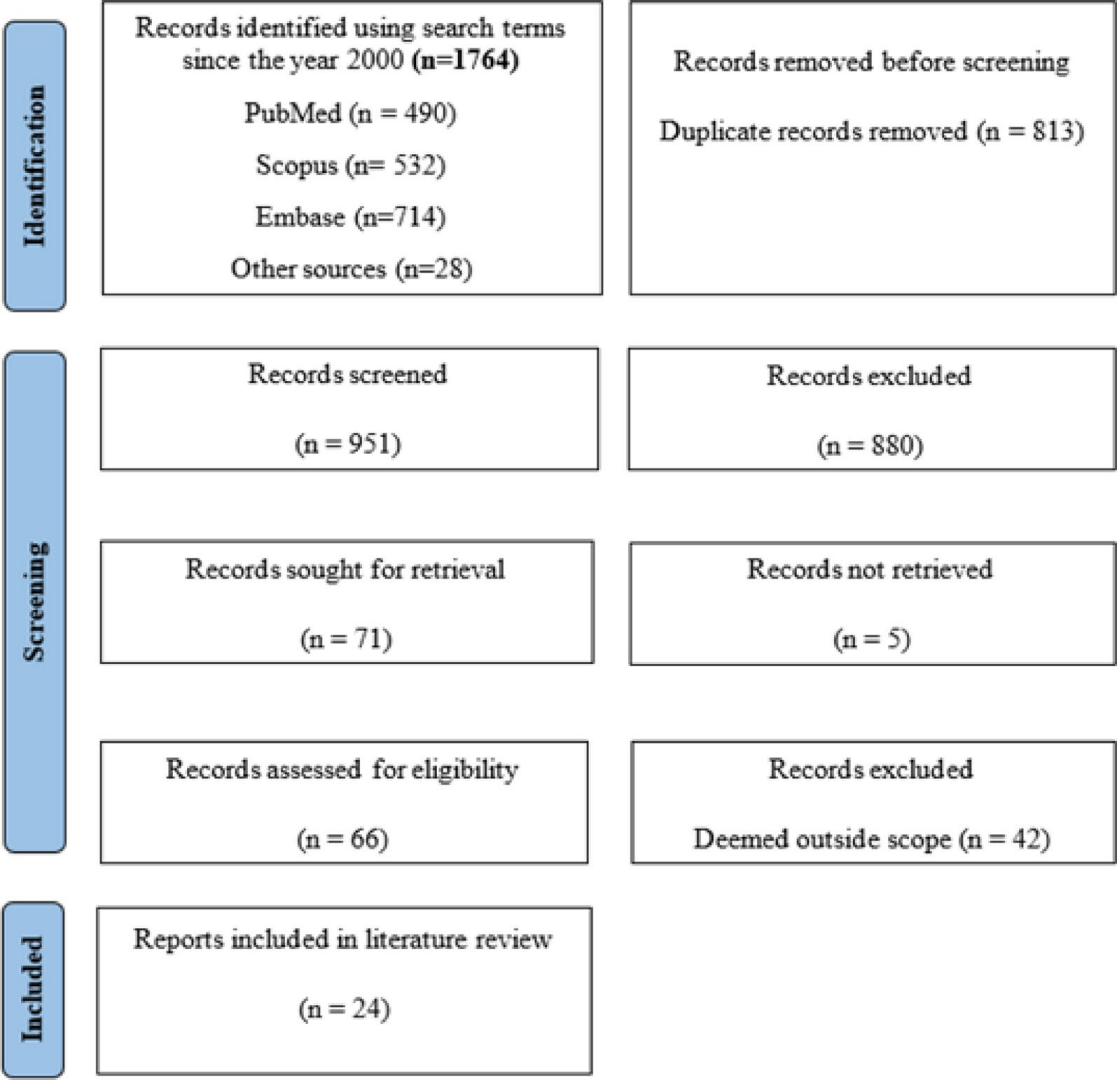
PRISMA-ScR flow diagram

The initial search across the databases generated 1746 results and 28 records from other sources were added to the review. The PRISMA Extension for Scoping Reviews (PRISMA-ScR) flow diagram outlines the study selection process (Fig. 1). After an initial screening of titles and abstracts, full texts were retrieved for records meeting the inclusion criteria or in cases where suitability was uncertain. If a study seemed to fulfil the inclusion criteria but data was insufficient or involved the wrong comparator, it was excluded. Full text records were reviewed by a second reviewer. This scoping review was completed with a final selection of 24 records upon which both reviewers agreed.

Inclusion criteria

- Papers written in English, published between 2005 and 2025 to ensure relevance to contemporary naloxone interventions and policies.
- Studies examining naloxone provision in incarcerated populations, including take-home naloxone on release, but excluding programmes for opioid users in the general community. This ensures the review focuses on interventions affected by prison-specific policies, staffing, security considerations and institutional structures Randomised controlled trials, cross-sectional studies, and studies reporting acceptability or feasibility of naloxone in carceral settings. This allows inclusion of both quantitative effectiveness data and qualitative insights into implementation
- Studies on perspectives or views of incarcerated individuals, healthcare professionals, or correctional staff on naloxone provision, to identify barriers, facilitators, and practical considerations for programme delivery

Exclusion criteria

- Studies examining community-only programmes not linked to incarceration, as they do not address prison-specific implementation factors
- Studies focusing solely on buprenorphine/naloxone (Suboxone). Although “naloxone” is part of these products and may appear in database searches, these interventions are for opioid substitution therapy rather than naloxone overdose-reversal programmes and are therefore outside the scope of this review
- Protocols without any preliminary results, conference abstracts and non-empirical studies, to ensure inclusion of studies reporting actual implementation, outcomes, or stakeholder experiences

### Stage 4: charting the data

Once all exclusion criteria were applied, the data from the remaining records were charted. We summarised them in a table format to allow for comparison and thematic analysis—Author(s), year of publication, journal, title, study design, population, aim of the study and major findings.

### Stage 5: collecting summarising and reporting results

Extracted data were collected, presented and reported in the results section (see Table 1). Key themes were identified using narrative synthesis as informed by Popay et al. [33].

**Table 1:** Records included in the review (n = 24)

References	Journal	Title	Study design	Population	Aim	Findings
Wakeman et al. [34]	Journal of Addictive diseases	Preventing Death Among the Recently Incarcerated: An Argument for Naloxone Prescription Before Release	Cross-sectional study	Rhode Island prisoners (n = 137)	Assess naloxone acceptability	72% interested in prescription, 90% interested in training
Cropsey et al. [35]	Journal of Opioid Management	Characterization of opioid overdose and response in a high-risk community corrections sample: A preliminary study	Cross-sectional study	Alabama community corrections (n = 478)	Assess overdose risk and naloxone interest	Three groups—opioid overdose, drug users but never experienced overdose and non-opioids Opioid OD group more willing to use naloxone; low prior use overall
Bird et al. [36]	Addiction	Effectiveness of Scotland's National Naloxone Programme for reducing opioid-related deaths: a before (2006–10) versus after (2011–13) comparison	Cohort study	Scotland (prisons and community) (n = 1970 (2006–10) & n = 1212 (2011–13))	Evaluation of national Take-home naloxone (THN) rollout	Overdose deaths within 4 weeks post-release reduced from 10 to 5%. 90% of naloxone kits are distributed to people at risk of opioid related overdose. Training provided by staff, pharmacists, voluntary workers and peer-trainers
Sondhi et al. [37]	Drug and Alcohol	Addressing perceptions of opiate-using prisoners to take-home naloxone: findings from one English region	Qualitative study	England (n = 142)	Assess perceptions and feasibility of THN programme	Poor knowledge and least usage of naloxone before training and confidence increased post-training. Barriers include stigma, lack of knowledge and misconceptions
Parmar et al. [38]	Addiction	Randomized controlled pilot trial of naloxone-on-release to prevent post-prison opioid overdose deaths	Randomized controlled trial	England (n = 218 across 16 prisons)	Evaluation of N-ALIVE trial RCT	Easy to use, self-administration, no significant difference on overdose reported between intervention and control arms, tracking post-release was challenging
Petterson et al. [39]	Harm reduction journal	Overdose prevention training with naloxone distribution in a prison in Oslo, Norway: a preliminary study	Cross-sectional study	Norway, Oslo (n = 31)	Evaluate awareness and knowledge	Over half unaware of naloxone’s effects and risks. Suggests peer-administered naloxone following brief educational interventions
Curtis et al. [40]	Harm Reduction Journal	Acceptability of prison-based take-home naloxone programmes among a cohort of incarcerated men with a history of regular injecting drug use	Cross-sectional study	Victoria, Australia (n = 400)	Assess THN training acceptability	High interest in training; 95% willing to use naloxone, 94% willing to resuscitate a friend using THN if trained and 91% willing to be revived by a training peer
Green et al. [41]	JAMA Psychiatry	Post-incarceration Fatal Overdoses After Implementing Medications for Addiction Treatment in a Statewide Correctional System	Retrospective cohort study	Rhode Island (n = 179)	Analyse fatal overdoses post-incarceration	MAT increased post-release; naloxone provision declined among deceased
Davidson et al. [42]	Addictive Behaviors	Documenting need for naloxone distribution in the Los Angeles County jail system	Cross-sectional study	Los Angeles county jail (n = 3781)	Acceptability of naloxone distribution	40% wanted training on how to recognise an overdose and respond using naloxone
Pearce et al. [43]	International Journal of Prison Health	An evaluation of Take Home Naloxone program implementation in British Columbian correctional facilities	Qualitative study	Two correctional facilities British Columbia Canada (n = 9 staff)	Evaluation of THN programme	Facilitators reported include nurse training process by repeating group-based, hands-on training sessions conducted by a member of the regional harm reduction program. Education and training on naloxone reported essential
Wenger et al. [44]	Journal of correctional healthcare	Overdose Education and Naloxone Distribution in the San Francisco County Jail	Prospective cohort study	San Francisco county jail (n = 637)	Evaluation of Overdose education and Naloxone distribution (OEND) program	67% opted to receive naloxone in their property, one-third used post-release to reverse overdose
Chambers et al. [45]	Health Promotion practice	Pilot Study of an Overdose First Aid Program in Juvenile Detention	Cross-sectional pilot study	New Mexico (n = 39 youth)	Assess programme acceptability	97% Narcan retention at follow-up; training completed by all
Moradmand-Badie et al. [46]	Drug Alcohol review	Feasibility and acceptability of take-home naloxone for people released from prison in New South Wales, Australia	Cross-sectional study	NSW, Australia (n = 105 released)	Acceptability of naloxone	Only 11 have completed THN training; most supported naloxone training and for naloxone supply at the time of release from prison
Reed et al. [47]	International Journal of Prison Health	Changes in overdose knowledge and attitudes in an incarcerated sample of people living with HIV	Quasi-experimental study	HIV patients in Philadelphia jail (n = 68)	Evaluating naloxone training impact	Participant knowledge and confidence to manage an overdose increased post-naloxone training
Showalter et al. [48]	Social Science & Medicine	Bridging institutional logics: Implementing naloxone distribution for people exiting jail in three California counties	Qualitative study	3 Californian counties (n = 34 stakeholders)	Feasibility of Jail based OEND programme	Barriers include lack of knowledge, misconceptions regarding opioid overdose or naloxone, and disparaging attitudes towards drug users. Identified penal vs harm reduction logics, need for interagency trust
McLeod et al. [49]	International Journal of Prison Health	Knowledge of the Good Samaritan Drug Overdose Act (GSDOA) and possession of a naloxone kit among people recently released from prison	Cross-sectional study	British Columbia Gates Peer Health Mentoring Programme (n = 137)	Knowledge of legal protections and possession of THN kit on release	28.3% overdose risk individuals did not have THN kit in possession. Higher proportion of individuals heard of legal protections like GSDOA, receiving naloxone training in prison and THN kit in possession on release
Tran et al. [50]	Journal of Substance Use and Addiction Treatment	Characterizing and combating the opioid epidemic in the Los Angeles County jail system	Prospective cohort study	Los Angeles County Jail System (187,528 inmates; 11,881 opioid users)	Evaluation of jail-based naloxone administration	94.6% survived unresponsive events on administration of naloxone, no adverse outcomes and no misuse reported
Oser et al. [51]	Health and Justice	Rapid jail-based implementation of overdose education and naloxone distribution in response to the COVID-19 pandemic	Prospective cohort study	Kentucky jails (n = 8 counties)	Feasibility of Healing communities OEND programme during COVID-19	Barriers include physical distancing requirements during COVID-19, staff stretched thin by COVID-19 protocols, unreliable internet and a lack of technology within jail infrastructure
Ricciardelli et al. [52]	International Journal of Prison Health	“We don’t even know where it’s kept”: exploring perspectives on naloxone administration by provincial correctional workers in Manitoba, Canada	Qualitative study	Manitoba, Canada (n = 257 staff)	Explore perspectives of the naloxone administration	Reported stigma or misconception doesn’t exist. lack of knowledge as major barrier. Confidence improved post-training
Sprunger et al. [53]	Health and Justice	Jail-based interventions to reduce risk for opioid-related overdose deaths: Examples of implementation within Ohio counties participating in the HEALing Communities Study	Cross-sectional study	Jails in Three Ohio counties (two urban and 1 rural)	Document the implementation of OEND programme	Strong collaboration between community and jail settings is important, technical support require for standing orders for naloxone, awareness and coordinating with stakeholder essential
Victor et al. [54]	Journal of Substance Use and Addiction Treatment	Naloxone vending machines in county jail	Cross-sectional study	Los Angeles County jail (n = 6 jails, 6 jail administrators)	Evaluation of naloxone vending machines rollout	63.5% increase in naloxone distribution. A good relationship between jail and community agencies is important
Adams et al. [55]	Health Policy and Technology	An impact evaluation of the Scottish take-home naloxone programme	Cohort study	Scotland (prisons and community) (n = 1970 (2006–10) & n = 6439 (2011–20))	Evaluation of national Take-home naloxone (THN) rollout	Overdose deaths within 4 weeks post-release reduced by 55% from 9.8% in 2006–10 to 4.4% in 2011–20. Cumulative reach of THN naloxone to individuals at risk of opioid overdose across the post-implementation period was 58%
Martin et al. [56]	Journal of Substance Use and Addiction Treatment	Evaluating public health vending machine rollout and utilization in criminal-legal settings	Cross-sectional study	Rhode Island corrections (n = 3720 items)	Evaluation of public health vending machine	221 naloxone kits accessed between 2022 and 2024 and hesitancy among individuals due to fear or stigma
Staton et al. [57]	Health and Justice	Overdose education and naloxone distribution among women with a history of OUD transitioning to the community following jail release	Prospective cohort study	9 Kentucky Jails (n = 703 women)	JCOIN project—THN programme evaluation	94% of women had a naloxone unit left in their property at the jail. Two-thirds are still interested in receiving naloxone reported at 3 months follow-up

### Stage 6: consultation

Although a formal consultation process was not undertaken, the preliminary findings and synthesis were shared with a multidisciplinary team comprising a general practitioner, a human rights advocate, naloxone lead of the HSE addiction services, and an addiction GP working within prison services. Their professional feedback and perspectives were used to refine the interpretation of findings and ensure the review’s relevance to clinical practice, harm reduction, and carceral health policy. This step aligns with Arksey and O’Malley’s recommendation to enhance the applicability and validity of scoping review findings through stakeholder engagement, even in a limited capacity.

## Results

Initial searches across the databases and other sources yielded 1764 results. Following duplicate removal and reviewing of titles and abstracts, 66 records were then followed with a full-text review. The search, identification and selection processes are summarised in the PRISMA ScR flow diagram (Fig. 1). The following 24 records were included in the final analysis.

### Study characteristics

The 24 records included were from seven countries including United States (n = 14), Canada (n = 3), United Kingdom (n = 4), Australia (n = 2) and Norway (n = 1), and adopted a range of methodologies. The study designs included cross-sectional studies (n = 11), cohort studies (n = 7), qualitative studies (n = 4), acceptability and feasibility study (n = 1, quasi-experimental study (n = 1) and randomised controlled trial (n = 1).

**Fig. 2:**
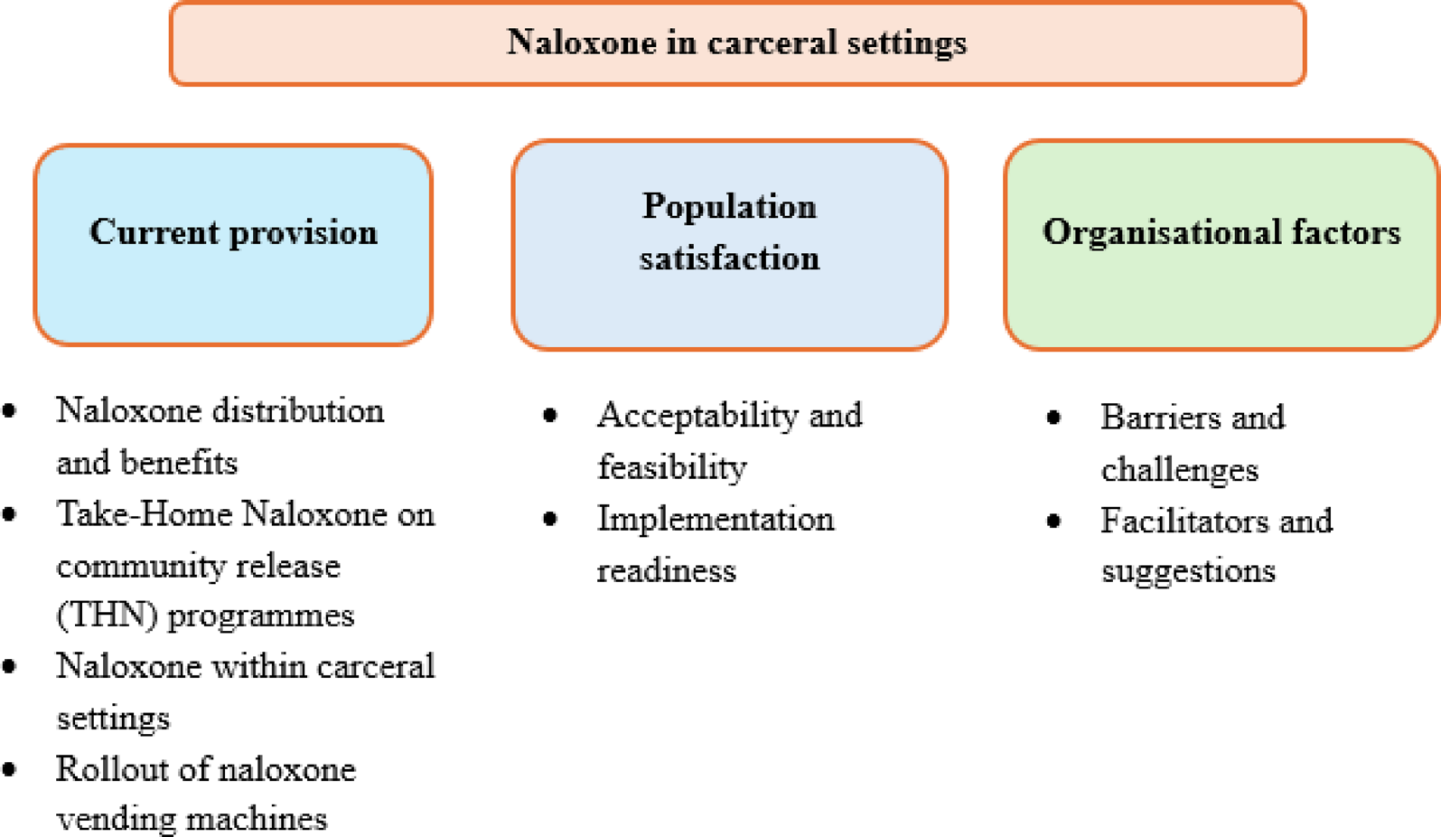
Themes identified on the review

The themes identified in this review are current provision (naloxone distribution and benefits, jail based take-home naloxone, naloxone within carceral settings and rollout of naloxone vending machines), population satisfaction (acceptability and feasibility, implementation readiness) and organisational factors (barriers and challenges, facilitators and suggestions) (see Figure 2).

#### Current naloxone provision

##### Naloxone distribution and benefits

Evidence from countries included in this review indicates that naloxone distribution in carceral settings is associated with reduced overdose deaths, increased overdose reversals with no or minimal adverse effects [36, 45, 50, 55]. Take-home naloxone (THN) programmes have been increasingly implemented in carceral settings, with 11 studies reporting the distribution of naloxone to incarcerated individuals upon their release, based on individual interest [37, 38, 44, 45, 47–49, 51, 53, 57]. However, only one study focused on naloxone administration occurring within the carceral setting itself [50].

##### Take-home naloxone on community release (THN) programmes

The THN programmes included in the review are the National Naloxone programme (NNP) in Scotland, the N-LIVE trial in England, the Overdose Education and Naloxone distribution (OEND) programme in Norway, San Francisco, New Mexico, California and British Columbia, the HEALing Communities programme in Kentucky and Ohio, and the JCOIN project in Kentucky [36, 38, 39, 44, 45, 48, 49, 51, 53, 55, 57]. The HEALing Communities Study implementation sites in Ohio and Kentucky refer to counties as ‘communities,’ within which multiple jails may operate; the studies focus specifically on jail-based interventions within these broader county-level areas. Overdose prevention in these settings includes a broad range of interventions such as cognitive-behavioural therapy, substance use treatment, training on overdose reversal and naloxone administration, as well as support services including parenting classes, case management, and linkage to community resources. These training sessions were delivered by nurses, pharmacists, voluntary sector workers and peer-trainers [36]. Studies also highlighted prisoners’ interest in peer-administered naloxone programmes [36, 39, 40].

In San Francisco, Wenger et al. reported that one-third of surveyed participants used naloxone to reverse an overdose following their release [44]. In Scotland, as an impact of naloxone distribution, overdose deaths within four weeks of prison release fell from 10% during 2006–2010 to 5% during 2011–2016, coinciding with the distribution of 36,000 naloxone kits over six years [36]. Similarly, among 804 women released from Kentucky jails between December 2020 and January 2024, naloxone was left with the belongings of 94%, and 70% reported receiving the naloxone upon release at the 3 month-follow up. An additional 4.7% requested to have it mailed to them [57]. The N-ALIVE randomised controlled trial in England reported 21% of participants reported administration of naloxone to themselves or someone else before the arrival of medical professionals, compared to only 9% in the control group [38]. The naloxone kit included a pre-filled syringe, an unscrewed plunger rod, and a safety-covered, sterile-packed hypodermic needle. Participants were instructed to assemble the syringe by attaching the plunger rod to the barrel and then administer the naloxone intramuscularly in the event of an overdose [38].

##### Naloxone within carceral settings

In Los Angeles County jail, Tran et al. documented the intranasal provision of naloxone between June 2018 and December 2020. A total of 184 kits were distributed to housing units, with correctional staff administering naloxone to 129 unresponsive individuals. Remarkably, 94.6% survived the incident, with no adverse outcomes or misuse reported [50].

##### Rollout of naloxone vending machines

The U.S. Centers for Disease Control and Provision’ Overdose Data to Action in collaboration with the Michigan Department of Health and Human Services installed naloxone spray vending machines at ten facilities in or near jail locations across Michigan. These machines were intended for returning citizens and visitors, not for in-prison distribution. This rollout led to a 63.5% increase in naloxone distribution with one facility going from zero to 1,000 distributed kits [54]. A similar initiative in Rhode Island, targeting corrections, probation, and parole locations, saw 221 kits accessed between May 2022 and February 2024. However, the evaluation highlighted hesitancy among some individuals due to fear or stigma associated with collecting naloxone near correctional settings [56].

#### Population satisfaction

##### Acceptability and feasibility

Several studies report high levels of acceptability and feasibility of naloxone provision among incarcerated individuals [57]. At the Bernalillo County Youth Services Centre in New Mexico, 97% of adolescents retained their Narcan kit at one-month follow-up, a rate that remained steady at three months [45]. In San Francisco, Wenger et al. reported that 44% of participants obtained a naloxone refill after release, with 32% using it to reverse an overdose and 12% giving it to someone unable to access it themselves [44].

Despite the positive outcomes, barriers remain. These include limited knowledge about naloxone, reliance on peer word-of-mouth, and fears such as experiencing “instant rattle”, a term describing sudden withdrawal symptoms triggered by naloxone use [37]. However, training and overdose education have proven effective in mitigating these concerns. Studies in incarcerated population in Philadelphia, England and Kentucky demonstrated significant improvements in knowledge and confidence in using naloxone following targeted training and education programmes [37, 47, 57].

##### Implementation readiness

Evidence from Rhode Island, Alabama, Victoria, and Los Angeles demonstrates high interest in naloxone prescription and THN among incarcerated individuals, particularly those who had never received naloxone previously [34, 35, 40, 42]. For example, Wakeman et al. found that despite naloxone not being available at the time in Rhode Island, incarcerated individuals expressed a strong interest in being prescribed naloxone and receiving training on overdose recognition, prevention, rescue, and naloxone administration [34].

However, several concerns reported include fears that naloxone training might introduce syringes or needles into the carceral setting in case of intramuscular naloxone, worries about being labelled as a drug user by prison staff and doubts about the effectiveness of naloxone itself [46]. Further barriers to implementation include significant knowledge gaps about how quickly naloxone takes effect, how long its effects last, need for a second dose if the first is ineffective, potential for re-overdose after naloxone use, fact that naloxone has a shorter half-life than many opioids and likelihood of naloxone inducing withdrawal symptoms [39].

Gender differences were also observed. Men were less likely to report that they would go to a pharmacy to obtain naloxone if recommended and were more likely to say that obtaining naloxone post-release was not a priority [46].

#### Organisational factors

##### Barriers and challenges

Several organisational and systemic challenges limit the effective distribution and use of naloxone in carceral settings and during the transition back to the community.

Under-provision, inconsistent access, stigma and misconceptions among the correctional staff remain significant issues [36, 41, 54]. A study in Rhode Island noted a decline in the number of naloxone kits dispensed at the point of release, 72 kits were distributed in the first six months of 2016 compared to only 35 in the same period of 2017. Meanwhile, a study of fatal opioid overdose deaths found that receipt of medication-assisted treatment (MAT) increased, while naloxone provision declined; however, the reduction in naloxone distribution could not be explained by changes in the population or by issues with provision [41]. In British Columbia, a THN study found that 28.3% of respondents who were considered at risk of overdose did not receive a kit at release. Additionally, 41.9% of those who received a kit reported that they did not perceive themselves to be at risk of overdose [49]. However, because the primary focus of the study was on the Good Samaritan law, it is unclear whether this finding reflects a true disconnect between perceived and actual risk. This highlights the importance of assessing both objective risk factors and individual perceptions when designing THN programmes in carceral settings.

In Scotland, THN was provided to 67% of recently released female prisoners but only 3% of male counterparts. Younger individuals under 35 were more likely to receive naloxone both in prison and the community, while older individuals and those with no recent injecting drug use history were less likely to be reached. Community provision was particularly low among people with a history of injecting drugs who had not injected in the past six months [36].

A study conducted during COVID-19 highlighted how physical distancing requirements, limited staffing capacity, unreliable internet, and outdated jail infrastructure hindered the continuation of naloxone training and distribution efforts during the pandemic [51].

At an organisation level, misconceptions and knowledge gaps among staff continue to pose barriers. Some believed naloxone required a prescription or misunderstood its safety and efficacy, fearing it could encourage continued drug use. Others did not trust its effectiveness or were unaware that naloxone has been safely distributed via standing orders since 2003 in states like California [48]. A common perception was that providing naloxone at release could act as an incentive to return to drug use, contradicting the harm reduction goals of the THN program. Additional challenges included identifying eligible individuals, securing ongoing engagement across facilities, and logistical problems such as coordinating naloxone kit distribution and training at release [37]. For example, among 804 women released from Kentucky jails between December 2020 and January 2024, naloxone was left with the belongings of 94%, yet only 70% reported receiving it upon release at the 3-month follow-up [57]. However, another study among correctional staff reported no stigma and misperceptions surrounding substance misuse but reported being not comfortable using naloxone, lack of medical training, low confidence in recognising overdoses, and uncertainty around when naloxone should be administered [52]. Some staff were unaware that it is generally safe to give naloxone even if an opioid overdose is uncertain. A training programme addressing these issues proved effective in increasing confidence and correcting misconceptions [52].

Other challenges include underreported usage of naloxone after transitioning to the community as they might lose the kit or give them away, or use them without reporting, making it difficult to track outcomes and evaluate programme effectiveness [38] (Table 2). This underscores a broader challenge, the persistent difficulty in ensuring naloxone reaches all who may benefit. While universal distribution could mitigate inequities inherent in selective provision, it presents considerable logistical and financial constraints. Nonetheless, in contexts where incarceration is closely linked to drug prohibition, providing universal access to naloxone may represent a reasonable and equitable approach to overdose prevention.

**Table 2:** Organisation-level barriers and facilitators on provision of naloxone in carceral settings

Barriers	Facilitators
• Under-provision of naloxone • Older PWIDs and people with no recent injecting drug use history were excluded in both prison and community • Inconsistent access • Stigma and misconceptions among staff • Limited knowledge about naloxone and difficulty identifying eligible individuals • Challenges transitioning from prison to community; underreported usage and inability to track programme effectiveness • Staffing shortages, unreliable technology, and outdated infrastructure	• Technical support for naloxone distribution through standing orders • Staff training programmes on naloxone use (including hands-on sessions) • Development of training materials and information on refill locations • Post-release harm reduction services and training • Training correctional staff to carry or store intranasal naloxone on site • Whole system approach, involving prison governor and all staff to reform harm reduction strategies • Resolving the conflict between penal logic and harm reduction logic • Strong collaboration between correctional facilities and community organisations • Stakeholder coordination and awareness building on naloxone policies • Partnerships with provincial pre-trial centres

##### Facilitators and suggestions

Several studies have identified key facilitators and practical recommendations to improve the implementation and uptake of naloxone programmes within carceral settings.

Research from Ohio and Los Angeles emphasised the importance of strong collaboration between correctional facilities and community organisations [53, 54]. Effective implementation requires not only technical support for standing orders that authorize naloxone distribution but also stakeholder coordination and awareness-building to foster understanding of local and state naloxone policies. This coordination can help reduce hesitancy and dispel misconceptions among correctional staff.

Sondhi et al. highlighted the value of a whole-system approach, suggesting that involvement from prison governors can significantly shift institutional culture and support harm reduction strategies [37]. Similarly, building interorganisational bridges or intermediaries that facilitate communication between incarcerated individuals and staff can help reconcile conflicting institutional logics, such as the punitive orientation of corrections (penal logic) and the public health goals of harm reduction (harm reduction logic) [48]. Other organisational level facilitators include strengthening nurse training programmes through repeated, group-based, hands-on sessions led by regional harm reduction teams, enhancing programme awareness via secondary advertising within prisons, partnering with provincial pre-trial centres to streamline promotion and recruitment once THN programmes are expanded across all facilities, developing clear, step-by-step training materials and shorter, more relatable instructional videos tailored to incarcerated populations, providing information on naloxone refill locations and post-release harm reduction services and offering basic education and training to correctional officers, including exploring the potential for them to carry or store intranasal naloxone on-site [43] (Table 2).

## Discussion

### Key findings

This review highlights the effectiveness, acceptability, and feasibility of naloxone use within carceral settings [37, 44, 45, 47, 57]. Naloxone has been shown to reduce overdose deaths and prevent the risk of overdose among incarcerated individuals [36, 45, 55]. While most individuals in prison express strong support for naloxone provision and training during their time in carceral settings, its implementation remains inconsistent and shaped by multiple barriers and facilitators [34, 35, 40, 42].

### Barriers—structural, cultural and operational challenges

#### Stigma and misconceptions

Stigma associated with drug use and naloxone remains prevalent among both incarcerated individuals and correctional staff [46, 48, 54]. Misconceptions that naloxone encourages substance use or signals tolerance of drug activity reflect deeper tensions between penal control and harm-reduction principles. These attitudes can suppress open discussion of overdose risk and inhibit staff engagement. Addressing stigma requires cultural as well as educational change reframing overdose prevention as integral to institutional health and safety rather than a discretionary public-health initiative.

#### Knowledge gaps and unequal access

Limited knowledge about naloxone and uncertainty in identifying eligible individuals continue to restrict uptake [46]. Older PWIDs and those with no recent injecting history are frequently excluded from training or kit distribution in both prison and community settings, despite evidence of elevated overdose risk post-release [36]. This selective approach underscores the need for explicit institutional policies that guarantee equitable access to naloxone across demographic and behavioural profiles.

#### Systemic constraints

Under-provision of naloxone, staffing shortages, unreliable technology, and outdated infrastructure have also constrained programme reach. Inconsistent supply chains and the absence of reliable data systems hinder the ability to monitor use and evaluate outcomes. These operational weaknesses make it difficult to demonstrate effectiveness, secure funding, or sustain institutional commitment.

### Facilitators—enablers of effective implementation

#### Training and education

Training programmes focused on overdose education and naloxone distribution have been shown to mitigate stigma and improve staff confidence [43, 52]. Practical, scenario-based training for correctional and healthcare staff combined with awareness materials on refill locations and post-release services can normalise naloxone within the broader emergency-response framework. Evidence from community settings shows that hands-on, repeated training yields the highest retention of overdose-response skills.

#### Peer and pharmacist involvement

Although few studies reported peer-to-peer naloxone administration within prisons, peer-facilitated and pharmacist-supported training models improved engagement and knowledge [35, 38, 39]. Scotland’s Peer Naloxone Supply Project demonstrated that peer educators can successfully distribute large numbers of kits while improving mutual trust between staff and prisoners [58]. Incorporating similar approaches could enhance reach and sustainability.

#### Whole-system approach

A whole-system approach involving prison governors, staff, and inmates is essential for successfully implementing naloxone programmes within carceral environments [37, 48]. This includes prison governors and senior administrators integrating naloxone into institutional policy frameworks and aligning overdose prevention with health and safety priorities, correctional and healthcare staff receiving structured overdose response training and being empowered to facilitate prisoner engagement and prisoners being involved in peer education, naloxone distribution and overdose awareness initiatives [37, 48].

Such an integrated framework recognises prisons as complex health ecosystems where leadership commitment, staff participation, and peer involvement are mutually reinforcing. Successful implementation would be characterised by consistent policy support, multi-disciplinary coordination, and continuous programme evaluation.

### Evidence gaps and implications

Despite growing recognition of naloxone’s value, evidence on in-prison administration and take-home naloxone remains extremely limited. Only three studies from the United Kingdom and one from Norway have examined take-home naloxone programmes on community release (no new programmes reported after 2017), and no studies have investigated naloxone administration within carceral settings [36–39].

The absence of evidence has critical implications as it remains unclear how institutional procedures, staff response capacity, and security considerations affect in-prison naloxone use. Similarly, the limited evaluation of take-home naloxone initiatives constrains understanding of continuity of care during the high-risk post-release period. These gaps hinder the development of best-practice models, delay policy standardisation, and may contribute to preventable overdose deaths.

Furthermore, there is a lack of research on naloxone provision among women prisoners, immigration detainees and within juvenile detention facilities worldwide [45, 57]. Tailored research is required to capture gendered and developmental factors influencing risk perception, healthcare access, and peer support within these groups. Further investigation should explore peer-to-peer naloxone use, comparative effectiveness of intranasal versus intramuscular delivery, and integration of naloxone with broader harm-reduction initiatives such as needle-exchange or opioid-agonist therapy.

### Comparing with international evidence

The findings of this review align with international evidence demonstrating that widespread naloxone distribution significantly reduces overdose mortality. In Scotland, overdose deaths dropped from 10 to 5% over six years following the introduction of a national naloxone distribution programme [36]. In the United States, community-based distribution prevented 352 opioid-related deaths and achieved 90% successful reversals among 16,429 distributed kits [59, 60]. A national survey conducted across Single State Agencies (SSAs), key bodies in the United States opioid response responsible for administering federal, state, and local funds to ensure service quality, promote best practices, and expand access to care found that all 45 SSAs had statewide naloxone distribution efforts, with 100% of SSAs using grant funds for naloxone distribution and 93% for naloxone training [61]. Similarly, Chicago’s OEND initiative achieved a 30% reduction in deaths after implementation [62].

Despite high feasibility of naloxone in incarcerated populations, awareness and perceived risk remain limited. A 2023 study among community pharmacists in Scotland, which found low risk perception and limited dose awareness among chronic non-cancer pain patients prescribed high-strength opioids [63]. Tatara et al. in Cook County (2020) which showed that jail-based naloxone distribution led to 24.4% of averted deaths post-release more than community only and combined models [64]. The importance of integrating jail- and community-based efforts is emphasised to ensure better outcomes and reduce duplication of services. This aligns with the findings of Hunt et al. which underscores the need for partnerships among state, community, and correctional stakeholders to expand access to this lifesaving, evidence-based intervention [65].

In Irish prisons, nurses are rostered 24/7 and have access to naloxone for use in medical emergencies; however, naloxone is not provided directly to prisoners during incarceration, and provision on release remains non-standardised. Recent overdose clusters, possibly involving synthetic opioids, underscore the urgency of expanding naloxone provision, implementing structured frameworks similar to Scotland’s framework for the supply of naloxone within prisons and Frankfurt University of Applied Sciences’s guidelines for naloxone provision upon release from prison and other custodial settings, and addressing the ongoing risks posed by emerging substances [66–69].

### Methodological considerations

While Horton et al. conducted a mapping review focused solely on take-home naloxone at release, this scoping review is the first to examine naloxone provision across carceral settings, including both in-prison administration and take-home distribution on release [70]. It also uniquely evaluates the acceptability of these interventions among correctional staff, healthcare workers and the incarcerated population, covering the period from the inception of naloxone programmes in 2000 through 2025. Although we used the Arksey and O’Malley’s [32] six-step methodological framework, a few limitations should be considered when interpreting the findings of this review. To minimise the errors, two reviewers identified relevant studies, and we adopted a comprehensive search approach, however, there is a possibility that not all publications relevant to the inclusion criteria were identified by the searches or databases used. We also did not assess the quality of studies, as the focus was on mapping the evidence. However, this scoping review captured the breadth of the topic naloxone in carceral settings.

### Implications for research, education and practice

Addressing stigma, misconceptions, and knowledge gaps among both correctional staff and incarcerated individuals is essential for the effective implementation of overdose prevention programmes and persistence of stigma, under-provision and fragmented governance suggests a need for national coordination. Adopting a unified policy framework would ensure that naloxone provision is viewed as a core custodial health service. Routine inclusion in emergency-response training, supported by technical infrastructure and supply chains, would normalise naloxone use. Collaboration between prisons and community organisations is critical for seamless transitions, ensuring that overdose education and access are sustained after release.

For Ireland specifically, the establishment of a standardised take-home naloxone scheme, integrated with existing harm-reduction programmes, could mitigate post-release mortality and address emerging synthetic-opioid threats. Future research should assess the feasibility and acceptability of prisoner-led naloxone administration, evaluate the effectiveness of peer-versus staff-led training models, examine continuity of naloxone access during the community re-entry period and explore integration with other harm-reduction measures.

Educationally, both correctional and healthcare staff should receive structured overdose-response training that incorporates stigma reduction and hands-on skill development. Embedding naloxone training into prison orientation and ongoing staff development could transform institutional culture and strengthen collective capacity for overdose prevention.

## Conclusion

Naloxone has emerged as a promising and life-saving intervention and public health measure within carceral settings. Evidence from international studies supports its role in preventing fatal overdoses and facilitating safer reintegration into the community. Using the barriers and facilitators identified in this review provides a blueprint for system-level reform. Effective implementation will depend on leadership engagement, staff empowerment, peer participation, and evidence-driven policy frameworks. Addressing the current evidence gaps particularly around in-prison use, gendered impacts, and post-release continuity will be essential to reducing preventable overdose deaths and positioning prisons as integral components of national harm-reduction systems.

## Data Availability

All the data associated with this study is available within the manuscript.
